# Application of online to offline teaching mode in the training of non-anesthesiology residents in the department of anesthesiology: a randomized, controlled trial

**DOI:** 10.3389/fmed.2024.1329538

**Published:** 2024-04-29

**Authors:** Yuan-yuan Zhao, Ting-ting Zhang, Ling-hui Li, Qian Liu, Li-juan Peng, Qi Wang, Wei Wang, Wan-you Yu

**Affiliations:** ^1^Department of Anesthesiology, Huainan First People’s Hospital, The First Affiliated Hospital of Anhui University of Science and Technology, Huainan, China; ^2^Department of Anesthesiology, The Affiliated Jiangning Hospital of Nanjing Medical University, Nangjing, China

**Keywords:** online to offline, teaching mode, residents, standardized training, anesthesiology

## Abstract

**Objective:**

To explore the effect of applying the online to offline teaching mode in the training of non-anesthesiology residents in department of anesthesiology.

**Trial design:**

The randomized controlled trial was performed on non-anesthesiology residents from Affiliated Jiangning Hospital of Nanjing Medical University.

**Methods:**

All selected residents were randomly divided into the traditional teaching group (Group T) and the online to offline teaching group (Group O) by the random number table method. Traditional teaching mode was used in Group T, while the online to offline teaching mode was used in Group O. The training period lasted for two months. At the end of the training, theoretical and clinical skills were assessed for all residents, and students’ satisfaction scores on teaching were investigated from the aspects of teaching mode, stimulating learning interest, improving learning process and teaching satisfaction. The teaching efficiency was compared and analyzed in the two groups.

**Results:**

In total, 39 cases in Group O and 38 cases in Group T were included in the statistical analysis. Compared with Group T, theory test scores, clinical skills test scores, and overall scores improved significantly in Group O (82.2 ± 8.1 vs. 91.3 ± 7.6; 85.1 ± 4.7 vs. 93.3 ± 5.4 and 83.4 ± 6.4 vs. 92.1 ± 6.7, respectively, *p* < 0.01). Compared with Group T, scores on teaching mode, stimulating learning interest, improving learning process and teaching satisfaction were higher in Group O (81.1 ± 6.9 vs. 93.7 ± 5.2; 83.6 ± 5.8 vs. 91.6 ± 6.4; 82.4 ± 5.3 vs. 90.9 ± 4.8 and 82.1 ± 5.9 vs. 92.1 ± 5.5, respectively, *p* < 0.01).

**Conclusion:**

The online to offline teaching mode can improve the level of professional theory and clinical skill operation, and teaching satisfaction of the non-anesthesiology residents in department of anesthesiology, thus improving the teaching effectiveness.

## 1 Introduction

Standardized training for residents is an important part of post-graduation education for medical students. With the increasingly refined division of clinical disciplines, although clinical specialists are familiar with the knowledge and skills of their major, they have some deficiencies in theoretical knowledge and clinical practice of related disciplines outside their major (1). Various monitoring theories and operation techniques, emergency treatment of various perioperative complications and pain treatment involved in clinical anesthesia work are the basic skills that clinicians should master (2). Therefore, it is necessary to explore the problems existing during the rotation of non-anesthesiology residents to anesthesiology department and propose corresponding solutions and countermeasures. These students’ understanding of anesthesiology only was limited in the medical school of surgery introduction to anesthesiology part, and training time in department of anesthesiology was short in domestic hospitals (no more than two months). There were many problems in the process of teaching, such as students’ lack of purpose, enthusiasm and initiative, also teachers’ lack of enthusiasm and guidance, even strict training rules and evaluation plans (3, 4). Therefore, compared with the traditional teaching by PowerPoint (PPT) and auxiliary skills operation, it is particularly important to stimulate these students’ interest and active their initiative in learning non-professional knowledge.

With the rapid development of information and network technology, and the continuous innovation of educational technology and means, the advantages of the mixed teaching mode combining online teaching with traditional offline teaching have become increasingly significant (5). The online to offline (O2O) mode first emerged in the field of e-commerce, which referred to the business model that used the online Internet as an offline trading platform to promote consumption and promotion (6). When the O2O mode was introduced into teaching, its connotation produced a qualitative change. “O2O Teaching Mode” was the integration of online and offline teaching, which used computer information, network technology and platform (7, 8). Studies have shown that using the O2O teaching mode in teaching of English (9), computer science (10) and medical students (11) have achieved good results such as the improvement of students’ learning enthusiasm, interest and academic performance. Therefore, the aim of this study was to investigate the effect of the application of O2O teaching mode in the training of non-anesthesiology residents in the department of anesthesiology, so as to provide reference for improving the training quality of these residents.

## 2 Methods

### 2.1 Ethics statement

The Consolidated Standards of Reporting Trials (CONSORT) recommendations (12) were followed in this study for the design and implementation of randomized controlled trials. Ethical approval for this study (2021-02-028-K01) was provided by the Institutional Ethics Committee of the Affiliated Jiangning Hospital of Nanjing Medical University. All participants involved were informed of the proposal and gave their written, informed consent.

### 2.2 Participants

Eighty non-anesthesiology residents were enrolled in this study, who were trained from May 4th 2021 to May 5th 2023 in department of anesthesiology, the Affiliated Jiangning Hospital of Nanjing Medical University. All residents were randomly divided into two groups by the random number table method: the traditional teaching group (Group T) and the online to offline teaching group (Group O), with 40 cases in each group. The traditional teaching method was used in Group T, while the online to offline teaching mode was used in Group O. Inclusion criteria: (1) Non-anesthesiology postgraduate students; (2) The training time in department of anesthesiology was two months; (3) The students had physician qualification certificate. Exclusion criteria: Failure to complete the study according to the prescribed training program.

### 2.3 Sample size and randomization

According to previous relevant studies (7, 11) and results of preliminary test, it was estimated that the final score of residents in group O was about 9.5 points higher than that of Group T, α =0.05, 1- β =0.8, 36 cases were required in each group, assuming that the shedding rate was 10%, and the sample size included in the initial screening was 40 cases in each group. Residents were randomly assigned to one of the two groups. Random tables were generated by computer. Eighty sealed envelopes were prepared by a statistician who did not participate in the study.

### 2.4 Study design

#### 2.4.1 Group O

① Online class: teachers published PPT, clinical operation videos and background materials related to the training course through the Chinese university Massive Open Online Courses (MOOC) platform before classes, and students were required to use flexible intelligent devices to complete independent learning before class. The online platform provided detailed information about the number of learners, the learning progress of the students and duration of study. After students ended their independent learning and examination, the platform provided evaluation and analysis of teachers’ input, so that students could have a preliminary understanding of their knowledge mastery, and enter offline classes with thinking and problems about the course content. ② Offline class: residents were taught by PPT once a week, mainly face to face to discuss the theoretical and operational content of the platform that was difficult for students to understand. The operation of skills was from clinical operation videos by online class, observation, teacher-assisted practice to independent practice (Table 1).

**Table 1:** Standardized training program for non-anesthesia residents in the department of anesthesiology.

Time	Content	Requirements
**First month**		
First day	Training of entering the department	① Understanding the basic workflow and system of anesthesiology department. ② Preliminary understanding of the relevant knowledge of the anesthesiology department. ③ Exercising face mask ventilation, endotracheal intubation and other operations by the simulated person.
First week	Subprofessional observation (general surgery, urology, obstetrics and gynecology, vascular surgery)	① Further getting familiar with the daily work process of the anesthesiology department. ② Following the frontline teacher to observe and learn anesthesia management.
Second week	Subprofessional observation (orthopedics, oncology, E.N.T., cardiothoracic surgery, emergency)	① Preliminary understanding of the monitoring of complex anesthesia and management. ② Following the frontline teacher to learn pre-operative assessment related to anesthesia.
Third week	Sub-specialty practice (general surgery, urology)	① Practicing face mask ventilation, endotracheal intubation, and laryngeal mask placement etc. ② Learning to independently complete the anesthesia management of patients with simple conditions.
Fourth week	Sub-specialty practice (obstetrics and gynecology)	① Further skilled and independently complete mask ventilation, endotracheal intubation, laryngeal mask placement and other operations; Preliminary understanding of intraspinal anesthesia. ② Following the frontline teacher for preoperative visit; Mastering the special physiology of the puerpera.
**Second month**		
First week	Sub-specialty practice (orthopedics, vascular surgery)	① Completing the mask ventilation, endotracheal intubation and other operations independently. ② Performing anesthesaesthetic management of patients with simple conditions independently.
Second week	Sub-specialty practice (oncology, E.N.T.)	① Completing the mask ventilation, endotracheal intubation and other operations independently. ② Performing anesthesaesthetic management of patients with simple conditions independently. ③ Preliminary learning to master difficult airway management.
Third week	Sub-specialty practice (cardiothoracic surgery)	① Completing mask ventilation, endotracheal intubation, arteriovenous puncture, EEG monitoring and other operations independently. ② Participating in, formulating and implementing postoperative analgesic treatment plan, and independently conduct anesthesia management of patients with complicated conditions. ③ Participating in the study of anesthesia management of critically ill patients in cardiothoracic surgery.
Fourth week	Sub-specialty practice (emergency)	① Participating in the emergency duty, and further learn to master the emergency and difficult airway management of emergency surgery. ② Learning about anesthesia assessment, preparation, induction and management. ③ Learning anesthesia management for critically ill patients and complicated patients with emergency surgery.
Last day	Examination	Theory test, clinical skills test, and satisfaction survey.

#### 2.4.2 Group T

Residents were taught by PPT twice a week, and followed the teacher in the daily clinical process. The operation of skills training was also followed with Table 1, but from learning by offline class, observation, teacher-assisted practice to independent practice. Additionally, to ensure the comparability with Group O, we converted the online study time of students in Group O into the offline self-study time for students in Group T. We played the same PPT and clinical operation videos, distributed the same materials and test volumes in the classroom where there was a teacher who ensured the same duration of study as recorded by MOOC in Group O and provided evaluation and analysis of students’ independent learning and examination.

### 2.5 Outcomes

#### 2.5.1 Main outcomes

Residents’ final examination scores. All students took offline theoretical and operational exams on the last day of training in the department of anesthesiology. The theoretical examination was based on the questions according to the Content and Standards of Standardized Training for Resident Doctors (Trial in China), including single choice, multiple choice, noun explanation and essay questions. The clinical skills assessment was carried out according to the Standard Scheme for The Clinical Practice Ability of Standardized Training of Residents (Department of Anesthesiology in China), including connection of conventional monitor, use of simple respirator, endotracheal intubation, single cardiopulmonary resuscitation and electrical defibrillation. The scores consisted of two parts: theory test score and clinical skills test score, accounting for 100 points, respectively. Final score = 60% theoretical score + 40% skill score.

#### 2.5.2 Secondary outcomes

Satisfaction score for clinical teaching. The clinical teaching satisfaction questionnaire was used to evaluate residents’ satisfaction with clinical teaching. The questionnaire score mainly included four items: teaching mode, stimulating learning interest, improving learning process and teaching satisfaction, each with a full score of 100 points. The satisfaction survey was conducted anonymously.

### 2.6 Statistical analysis

Data analysis was performed by the SPSS (version 25.0, SPSS Inc., Chicago, IL, United States). Continuous variables were presented as mean ± standard deviation (SD), and comparisons between groups were performed by an independent sample *t*-tests. Categorical variables were presented as frequency, and comparisons between groups were performed using the χ^2^ test. A *p*-value <0.05 was considered to be statistically significant.

## 3 Results

### 3.1 Residents recruitment

**Figure 1:**
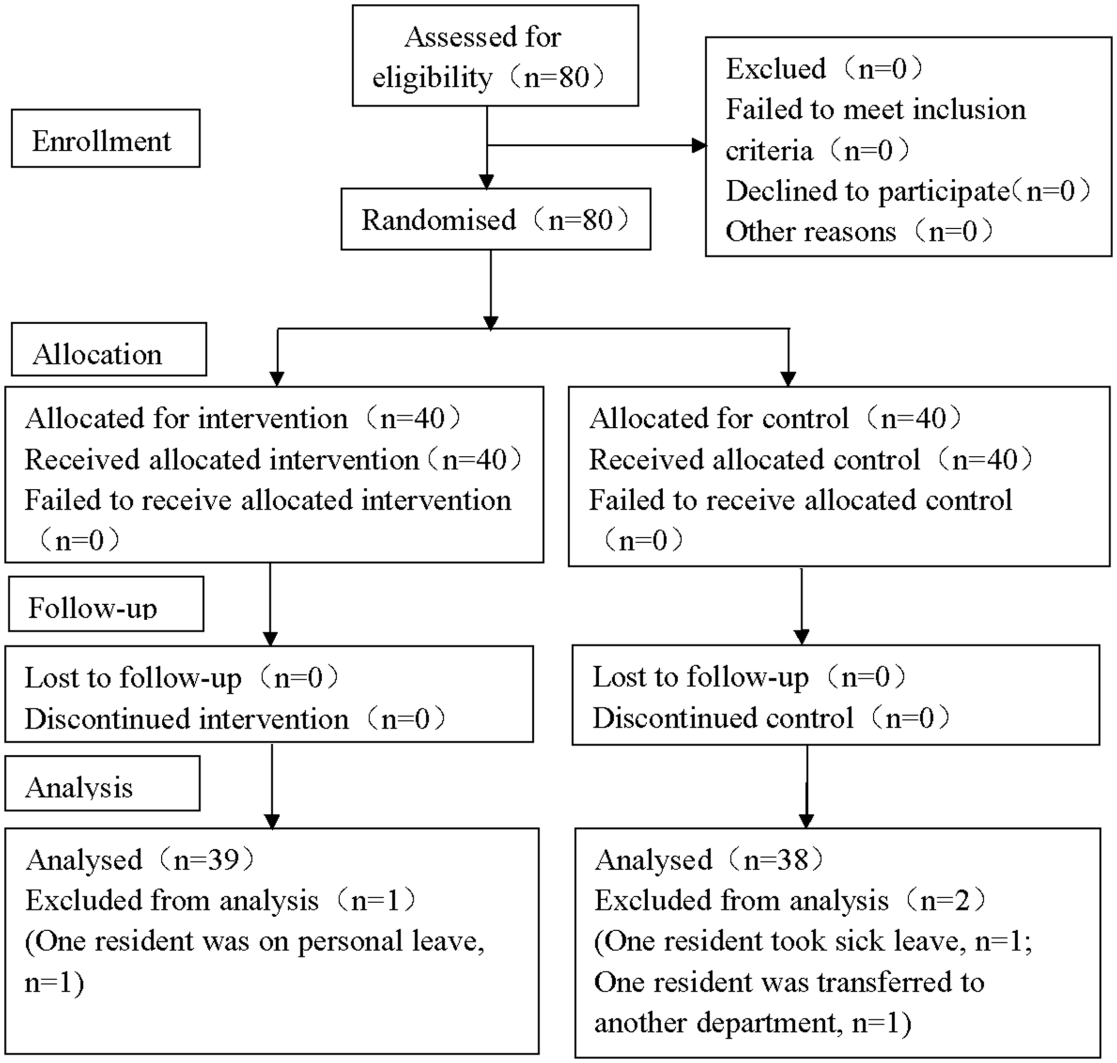
CONSORT flow diagram. In the study, a total of 80 participants were enrolled initially and three residents were excluded during the trial. Finally, there were 77 residents included in the statistical analysis (39 in Group O and 38 in Group C, respectively). CONSORT: the Consolidated Standards of Reporting Trials.

In this study, 80 residents were initially screened, and 3 of them were excluded (One resident was excluded for personal leave in Group O, while one was excluded for sick leave and another for transferred to other departments in Group T). In total, 39 cases in Group O and 38 cases in Group T were included in the statistical analysis (Figure 1).

### 3.2 Baseline characteristics of the two groups

The non-anesthesiology residents referred in this study majored in general surgery, orthopedics, emergency, critical care medicine, E.N.T., urology, oncology, cardiothoracic surgery and obstetrics and gynecology, respectively. There was no significant difference in age, gender, major, and type of postgraduates between group O and group T (*p* > 0.05) (Table 2).

**Table 2:** Comparison of baseline characteristics in the two groups (*n* = 77).

Characteristic	Group O (*n* = 39)	Group T (*n* = 38)	*p-*value
^a^Age (years), mean ± SD	25.3 ± 3.1	24.8 ± 3.6	0.826
^b^Gender (*n*)			
Male	22	20	0.318
Female	17	18	0.532
^b^Major (*n*)			
General surgery	6	5	0.916
Orthopedics	5	6	0.528
Emergency	6	5	0.916
Critical care medicine	3	4	0.428
E.N.T.	3	4	0.428
Urology	4	3	0.787
Oncology	5	3	0.624
Cardiothoracic surgery	3	3	0.998
Obstetrics and gynecology	4	5	0.722
^b^Type of postgraduates (*n*)			
Directed education	23	21	0.668
Socialized recruitment	16	17	0.325

SD, Standard deviation; E.N.T., ear, nose, and throat.

a Comparison based on an independent sample *t*-tests.

b Comparison based on χ^2^test.

### 3.3 Test score of the two groups

Compared with Group T, theory test scores, clinical skills test scores, and overall scores improved significantly in Group O (*p* < 0.01) (Table 3).

**Table 3:** Test score of residents in the two groups (mean ± SD, score).

Group	Theory test	Skills test	Total scores
Group O (*n* = 39)	91.3 ± 7.6	93.3 ± 5.4	92.1 ± 6.7
Group T (*n* = 38)	82.2 ± 8.1	85.1 ± 4.7	83.4 ± 6.4
*p-*value^a^	**0.004**	**0.006**	**0.004**

SD, Standard deviation; *p*-value significant (i.e., <0.05) indicated in bold.

a Comparison based on an independent sample *t*-tests.

### 3.4 Teaching score of the two groups

Compared with Group T, scores in teaching mode, stimulating learning interest, improving learning process and teaching satisfaction were higher in Group O (*p* < 0.01) (Table 4).

**Table 4:** Score of teaching in the two groups (mean ± SD, score).

Group	Teaching mode	Stimulation of learning interest	Improvement of learning process	Teaching satisfaction
Group O (*n* = 39)	93.7 ± 5.2	91.6 ± 6.4	90.9 ± 4.8	92.1 ± 5.5
Group T (*n* = 38)	81.1 ± 6.9	83.6 ± 5.8	82.4 ± 5.3	82.1 ± 5.9
*p-*value^a^	**0.002**	**0.005**	**0.004**	**0.002**

SD, Standard deviation; *p*-value significant (i.e., <0.05) indicated in bold.

a Comparison based on an independent sample *t*-tests.

## 4 Discussion

With the development of medicine, the scale of hospitals is constantly expanding, and the division of clinical disciplines is becoming refined. Various specialties pay more and more attention to the cultivation of the theory and skill operation of their professional doctors, but the learning of other interdisciplinary theories and skills are still insufficient. The department of anesthesiology of our hospital also undertakes a large number of standardized training tasks for non-anesthesiology residents every year, including directed education and socialized recruitment of postgraduates. These students are mainly involved in a variety of surgery-related majors, such as general surgery, orthopedics, emergency department, intensive care medicine, E.N.T., obstetrics and gynecology, etc. As residents in anesthesiology, we used to use traditional teaching by PPT and “hand-in-hand” operation training for non-anesthesiology residents, which are difficult to stimulate their interest in learning and often lack active initiative because of insufficient understanding of anesthesiology related theories and operations (13). Most of them reflected that the knowledge in class was difficult to understand, and the actual operation was also very passive and difficult to accept. Therefore, this study adopted the O2O teaching model with preview, talking and interaction online outside the classroom. The results suggested that this teaching model improve the teaching effectiveness to non-anesthesiology residents in department of anesthesiology.

### 4.1 Traditional teaching in department of anesthesiology

Traditional teaching methods are relatively simple in department of anesthesiology. The main body of teaching were teachers, who only regarded students as the container to accept knowledge, and to some extent ignored the existence of students as the subject of learning. Students only took the test for the purpose and mechanically memorized the knowledge points in the traditional teaching mode. In the study of Duan et al. (14), they compared the effects of an online teaching mode on WeChat platform with the traditional teaching model on learning outcomes of anesthesiology residents during the COVID-19 outbreak. The results showed that the examination performance, clinical thinking, communication skills, learning interest and self-learning ability of residents in the group of traditional teaching model were worse than those in the group of online teaching mode. However, online teaching alone cannot meet the needs of residents’ practical skills and face-to-face communication with teachers and patients. Thus, online to offline teaching mode was designed in this study. Additionally, the integration of teaching and information technology was poor used in traditional teaching mode. At present, the use of information technology in the department of anesthesiology often stayed in PPT, after-class paper examination and questionnaire survey. Some hospitals or teachers were still in the stage of multimedia teaching and “face-to-face” teaching, and they could not flexibly use various online teaching platforms, WeChat public accounts and other media. In the study of Huang et al. (15), they used WeChat public platform for education in anesthesiology residents. The results suggested that residents in the WeChat group perform significantly better on assessments than those in the traditional group regarding theoretical knowledge scores, operational skill scores and overall scores, and the questionnaire results indicated that the degree of satisfaction of the residents and teachers in the WeChat group was significantly higher than that in the traditional group. In contrast, the MOOC platform was used in the present study which was more versatile and the most widely used platform for teaching in China (16). Besides, residents’ feedback to the class was not optimistic in the traditional teaching mode (17). Some residents had not prepared before class and knew nothing about the content of teaching. It was difficult to understand the professional knowledge points. Therefore, they can not pay attention in class and thought the course was boring.

### 4.2 Classification of O2O teaching mode

According to the differences of curriculum design idea, the current O2O teaching modes were as follows. (1) The O2O teaching mode based on the traditional classroom idea was reported by Daulatabad et al. (18). In this mode, online learning was only an auxiliary and supplement to the offline traditional classroom. Teachers can upload teaching content and expand knowledge online for students’ preparation before class, consolidation and expansion after class. Offline classroom was still based on the students’ preparation before class. This type of teaching mode can be used for courses with strong theory and difficult for students’ self-study. (2) The O2O teaching mode based on the concept of flipped classroom reported by Zhang et al. (19). Students mainly chose courses and self-studied through online platform. They can also interact online through social network, participate in offline class regularly and conduct collaborative learning. This teaching mode can be used for easy self-study courses. (3) The O2O teaching mode based on the mixed teaching idea reported by Ding et al. (20). This mode divided the courses into easy and difficult content. Students studied easy content online by themselves, and made discussion and practice by offline face-to-face class. The difficult content was mainly learned by offline classroom lecture, while the online class was mainly for practice and consolidation. Considering the characteristics of anesthesiology and the correlation between different majors, the third O2O teaching mode was adopted in this study. Residents learned easy content online, and made discussion of difficult content in offline class. The results of this study showed that this mode improved the learning motivation of residents and the effect of teaching.

### 4.3 Advantages of the O2O teaching mode

Teaching resources were effectively utilized in the O2O teaching mode. In traditional teaching mode, many high-quality teaching resources on the internet, such as videos, animation and so on, were often unable to be used due to limitation of conditions. The study of Shi et al. (21) showed that teachers could directly send these resources to students through links and other forms in the O2O teaching mode, and save time and effort, which improved teachers’ work efficiency, so as to concentrate on polishing better course resources. Secondly, the time and space for students’ study were greatly expanded. Students can only listen in the classroom during class time in traditional teaching mode. Most of the O2O teaching mode was online education. Students can learn at any time with only a computer or a mobile phone, which was conducive to students to make better use of the fragmented time (22). However, online teaching did not completely replace classroom teaching. Thus, in our study, residents in Group O were also taught by PPT in Offline class once a week, mainly face to face to discuss the theoretical and operational content of the platform that was difficult for students to understand, which can improve students’ interest and efficiency in learning. The results of this study also showed that the theory test scores, clinical skills test scores, and overall scores improved significantly in Group O. In addition, the interaction between teachers and students was enhanced in the O2O teaching mode, which could improve the quality of teaching. According to the results of Asfhar et al. (23), teachers can learn students’ preview, review, homework completion, participation in discussion, online examination and other conditions through background data in O2O teaching mode, and students can also see teaching resources and data on the platform and become teaching supervisors. Therefore, teachers and students can communicate and interact online, score each other, which will be conducive to the establishment of a harmonious teacher-student relationship. So that in the present study, students had significantly higher scores of teaching mode and teaching satisfaction in Group O.

### 4.4 Limitations of the O2O teaching mode and this study

Mobile phones were one of the important learning tools in O2O teaching mode. Some students played mobile games in class, which might have a negative impact on learning (24). In this study, we increased the frequency of answering questions online and offline in class. Students who played mobile phones could not answer questions in time, which might lead to low scores in class, thus promoting students to improve their learning concentration. In addition, the function construction of the course platform was also very important. If the mobile phone was locked after the students’ scanning the code and entering the classroom, it could only be synchronized with the teacher’s mobile phone or computer. Study of Zhao et al. (25) showed that the extensive use of O2O teaching mode also brought great pressure to students.

Due to the fact that subjects of this study were non-anesthesiology residents and different training methods of medical students in China were trained in different ways, the examination methods in this study were not based on the standardized training examination for anesthesiology residents in the United States. However, there was no unified international standard for the standardized training of non-anesthesiology residents in the department of anesthesiology. In our study, the training of non-anesthesiology residents followed the same domestic standards including basic theory, knowledge and skills according to anesthesiology residents, which did not affect the evaluation and promotion of online to offline teaching mode. In addition, due to the large number of course chapters and automatic evaluation scores on the MOOC platform, the scores of students’ online learning and evaluation were not counted in this study, but the final theoretical and operational examination scores on the last day of training in the department of anesthesiology were taken as the main outcomes. Finally, though it was difficult to achieve double blinding in this study, we ensured those who scored the test and the questionnaire did not know the grouping.

## 5 Conclusion

In conclusion, this randomized control trial shows that application of online to offline teaching mode in non-anesthesiology residents in department of anesthesiology can improve the level of anesthesia-related professional theory and clinical skill operation in these students, and their satisfaction with teaching mode, so as to improve the teaching effectiveness.

## Data availability statement

The raw data supporting the conclusions of this article will be made available by the authors, without undue reservation.

## Ethics statement

Ethical approval for this study (2021-02-028-K01) was provided by the Institutional Ethics Committee of the Affiliated Jiangning Hospital of Nanjing Medical University. All participants involved were informed of the proposal and gave their written, informed consent.

## Author contributions

Y-yZ: Data curation, Funding acquisition, Methodology, Writing – original draft. T-tZ: Methodology, Software, Writing – original draft. L-hL: Conceptualization, Investigation, Writing – original draft. QL: Investigation, Writing – original draft. L-jP: Investigation, Writing – original draft. QW: Conceptualization, Investigation, Writing – original draft. WW: Project administration, Supervision, Writing – review & editing. W-yY: Data curation, Writing – review & editing.
